# Treatment patterns and real world clinical outcomes in ER+/HER2- post-menopausal metastatic breast cancer patients in the United States

**DOI:** 10.1186/s12885-017-3379-1

**Published:** 2017-06-02

**Authors:** Giovanni Zanotti, Matthias Hunger, Julia J Perkins, Ruslan Horblyuk, Monique Martin

**Affiliations:** 1 Mapi, Konrad-Zuse-Platz 11, 81829 Munich, Germany; 2 Pfizer Inc., 235E 42nd, New York, NY 10017 USA; 3 Mapi, Beaufort House, Cricket Field Road, Uxbridge, UB8 1QG UK

**Keywords:** Post-menopausal metastatic breast cancer, Treatment patterns, Physician survey, ER+/HER2

## Abstract

**Background:**

With several new therapies becoming available, treatment of metastatic breast cancer (mBC) is evolving. The objective of this study is to describe patient characteristics, treatment patterns and real-world clinical outcomes in post-menopausal women with ER+, HER2- mBC and to obtain insight into patient outcomes and potential unmet needs with current therapies.

**Methods:**

The current study is a physician survey followed by a retrospective chart review of patient medical records by physicians in the US between March and April 2015. One hundred three physicians were asked to complete an online survey aiming to understand their satisfaction and expectations with current available treatments and potential areas of unmet need for mBC patients. Medical records from 178 females were extracted for the chart review. Using these data from medical records, patient characteristics and treatment patterns were analyzed descriptively. Time to progression (TTP) on first line, and progression-free survival (PFS) on second and third line of therapy were analyzed using the Kaplan-Meier method.

**Results:**

Sixty-seven percent (*n* = 119) of patients had metastatic disease at initial diagnosis of breast cancer. Mean age at chart data extraction was 65.8 (SD: 9.4) years. Aromatase inhibitors (AIs) were prescribed for 58% and around 13% of patients in first line and second line, respectively. Chemotherapy was prescribed to 14% in first line and 31% in second line. Median TTP on first line therapy was 12 months for patients receiving AIs as compared to 7.9 months for patients receiving chemotherapy. Across all treatment lines, bone pain and fatigue were reported as the main symptoms associated with disease progression which had an impact on patient quality of life. Physicians expressed that prolonging life was deemed the most important treatment goal, followed by preservation or improvement of quality of life.

**Conclusion:**

In this study the majority of patients received endocrine therapy as first line treatment and current therapies still resulted in a short time to progression in first line. Results from the chart review and the physician survey highlight a quantitative unmet need for more effective treatments which delay disease progression and improve survival outcomes while maintaining quality of life.

## Background

Breast cancer (BC) is the most common cause of cancer death in women worldwide and estimated to be responsible for almost 460,000 deaths in 2008 [1]. Estimates from the United States for 2015 show that breast cancer accounts for 29% of all new cancers diagnosed and 15% of all cancer deaths in women [2, 3]. When diagnosed in early stages, treatment of BC is generally more effective resulting in a 5-year overall survival rate of 99% for stage I (localised stage) and 85% on average in regional stage, compared with only 25% for the metastatic stage IV [2]. However, early stage BC can recur and it is estimated that 20 to 30% of all patients diagnosed with early stage BC will eventually progress to metastatic disease over a lifetime [4]. Metastatic breast cancer is when breast cancer has spread beyond the breast and local lymph nodes under the arm to other areas of the body. The most common sites of metastases are the bones, lungs, liver and brain.

Approximately 6–10% of new breast cancer cases are diagnosed initially at stage IV or mBC [5] and it has been estimated that 155,000 Americans are currently living with mBC [6]. According to the 2008 American Society of Clinical oncology (ASCO) symposium report, the median survival rate after diagnosis of mBC was three years and no statistically significant improvement has been established since then [7, 8].

The majority of diagnosed breast cancers is Estrogen receptor-positive (ER+) and Human Epidermal Growth Factor Receptor 2 negative (HER2-). Endocrine therapy is the major treatment for ER+ and HER2- metastatic breast cancer [9]. In the last two decades, the third generation of aromatase inhibitors anastrozole, letrozole and exemestane have become the standard hormonal treatment for post-menopausal women in both advanced and early disease [9]. The efficacy of these compounds in terms of response rates in first line metastatic patients are up to 40% with all initial responders eventually developing resistance over time, meaning that there is an ongoing need in this population [10].

According to the National Comprehensive Cancer Network (NCCN) guideline, it is recommended to continue endocrine therapy after progression with a first endocrine agent, unless there is significant visceral burden or rapid progression of disease, where in this case chemotherapy is recommended [11]. Other endocrine therapies options include selective oestrogen receptor modulators like tamoxifen or selective oestrogen receptor degraders like fulvestrant.

However, real world treatment patterns and outcomes among patients with ER+, HER2- mBC are still not well characterized. A literature review by Boswell et al. [12] examined disease burden and treatment outcomes in second-line therapy of patients with ER+ advanced breast cancer. The authors concluded that there is insufficient evidence on effectiveness outcomes to quantify the unmet need in ER+ patients, and this gap warrants further research. Swallow et al. [13] conducted an analysis of MarketScan databases of patients with HR+, HER2- mBC between 2002 and 2012. They found that most patients initiating treatment with endocrine therapy (ET) received only one line of ET before discontinuation or transition to chemotherapy. Gaps in knowledge remain despite the availability of recent chart review studies in HR+, HER2- mBC [14–16]. A better understanding of patient characteristics, real world variations in treatment and their impact on clinical outcomes is needed to identify limitations of currently available therapies and patient needs.

The objective of this study is to describe patient characteristics, clinical outcomes observed in real-world as well as identification of aromatase inhibitors early non responder’s characteristics in post-menopausal women with ER+, HER2- mBC and to obtain insight on potential unmet needs in these patients.

## Methods

### Data source

Our study had two distinct components: a cross-sectional physician survey and a retrospective medical record review conducted by participating physicians between March and April 2015. Physicians specializing in medical oncology or hematology/oncology and treating patients with post-menopausal ER+, HER2- metastatic breast cancer were invited to participate from a US online physician panel. Physicians were eligible for the survey and the chart review if they personally treated 12 or more ER+, HER2- metastatic breast cancer patients within the last six months. Also, physicians were required to provide informed consent and to have been practicing medicine for the treatment of ER+, HER2- mBC patients for between two and thirty years. To achieve a sample representative of physicians treating mBC in the US, soft quota restrictions were applied for the region where physicians practice and approximately 60% of sites were required to be community-based practices.

All potential physician participants were screened for study eligibility using a standardized screening questionnaire developed for the study. No more than two physicians were allowed to be grouped per practice. Eligible physicians were asked to participate in an online survey including 25 questions on physicians’ perception of quality of life among patients ER+, HER2- mBC, physicians’ satisfaction with currently available treatments and potential areas of unmet need, and physician and patient interactions and dialogue. The survey was pilot-tested on three physicians and minor changes were made to the survey to reflect their comments. After completing the online survey, physicians were asked to extract individual patient data from medical records of two randomly selected patients and fill out an online case report form. Only de-identified data from the charts were abstracted and Institutional Review Board (IRB) approval was obtained for both the physician survey and the patient medical record data extraction components (Schulman IRB number 201500093). Research was performed in accordance with the Declaration of Helsinki.

### Patient selection

Records of female patients were eligible for chart data abstraction if they had a confirmed post-menopausal status per local practice guidelines at time of mBC diagnosis, had a confirmed diagnosis of metastatic breast cancer based on histological or cytological findings and had confirmed ER+ and HER2- BC per local practice guidelines. Furthermore, patients had to have received care from the same physician from diagnosis of mBC to the last available encounter in the medical record and had to have completed at least 2 lines of breast cancer therapy in the mBC setting between January 1, 2008, and March 1, 2014. This means that patients that died during first-line therapy or before initiation of second-line therapy could not be enrolled in the study. Completion included completion of prescribed treatment, disease progression, or discontinuation of treatment due to adverse events, loss to follow-up, patient request, or death. Patients were not eligible for the chart review if they had evidence of other concurrent malignancy, except adequately treated non-melanoma skin cancer or other noninvasive (in situ) neoplasms at the time of diagnosis of ER+, HER2- metastatic breast cancer. Patients who participated in a clinical trial or other interventional study related to breast cancer for any treatment in the metastatic setting were not eligible for the study either. Participants of observational studies or adjuvant clinical trials were allowed. A quota for survival status was applied to the selection of patients to ensure that 80% of patients selected were still alive at the date of data abstraction.

### Study outcomes

Chart data abstracted by the treating physician included information on demographic characteristics, disease history, treatments received by line of therapy, start and stop dates of the therapies, and reasons for treatment discontinuations. Primary reasons for discontinuation included – amongst others – completion of treatment as planned, disease progression, drug resistance, toxicities/side effects, or death. Time to disease progression on first-line therapy was defined as the time from the start of the therapy to the date of documented disease progression. Patients who completed first-line treatment as planned or who discontinued treatment for reasons other than disease progression were censored at the day of treatment completion or treatment discontinuation, respectively. As inclusion criteria required having completed at least two treatment lines, no deaths were observed during first line therapy. However, as some patients died while on second or third line therapy, progression-free survival (PFS) rather than TTP was analyzed for second and third line treatments. PFS on second and third line therapy was defined as the time from start of treatment to the date of documented disease progression or death. Patients who completed second or third line treatment as planned or who discontinued treatment for reasons other than disease progression were censored at the day of treatment completion or treatment discontinuation. Overall survival (OS) was defined as time from start of first-line treatment to death from any cause. For PFS and OS, patients without an event were censored at their chart abstraction date.

### Statistical analysis

All statistical analyses were descriptive in nature. Summary statistics were calculated to describe physicians’ responses in the survey and to describe demographics, clinical characteristics, and treatment patterns of patients from the chart review study. For continuous data, the mean, standard deviation and median are presented. For categorical data (including yes/no categories), the frequency and percentage in each category are presented. Analyses were stratified by line of treatment and type of treatment received where applicable. Time-to-event endpoints such as TTP on first-line therapy, PFS on second or third line therapy or OS were analyzed using Kaplan-Meier methods to appropriately take into account censored observations.

To explore the potential unmet need of patients receiving aromatase inhibitors who had an early treatment discontinuation, further bivariate analyses in this subgroup were conducted. For these analyses, early treatment discontinuation was defined using a cut-off of five months. Reasons for treatment discontinuation included progression, death, drug resistance or toxicities/side effects.

## Results

A total of 510 physicians were contacted through the online panel. Of those, 130 physicians were screened out because they did not meet inclusion criteria, and 277 physicians did not successfully complete the survey. A total of 103 physicians completed the survey and abstracted chart data from 178 post-menopausal patient medical records with confirmed ER+/HER2- mBC.

### Chart review

#### Patient characteristics

Of the 178 patients with confirmed metastatic disease and for whom data was extracted 119 (66.9%) had metastatic disease at initial diagnosis of ER+ HER2- breast cancer (Table 1). Eleven percent were initially diagnosed at stage IIIA, IIIB or IIIC, while 40 (22.5%) patients had a history of early disease. The mean age at chart data extraction was 65.8 years. Distant metastases were most common in the bone (73.0%; *n* = 130) followed by lung/pleura (36.5%; *n* = 65), lymph nodes (32.0%; *n* = 57) and the liver (21.4%; *n* = 38). The mean age at progression to metastatic disease was 62.9 years. Most patients (89.3%, *n* = 159) had an Eastern Cooperative Oncology Group (ECOG) status of 0 or 1 at the time of diagnosis of mBC. For the 65 patients (36.5%) with a history of adjuvant treatment, median duration of adjuvant treatment was 36 months. Main reasons for stopping earlier, non-metastatic therapy were successful completion of planned treatment course (43.1%) and progression to metastatic disease (41.5%).

**Table 1:** Patients characteristics – from chart review

Variable	Level	*N*	Mean	SD
Age at data extraction or death		178	65.83	9.35
Age at initial BC diagnosis		178	61.49	9.69
Age at progression to metastatic disease		178	62.85	9.39
			*n*	%
Stage at diagnosis	Early (Stage IA, IB, IIA, IIB)		40	22.5%
	Limited Regional (Stage IIIA)		8	4.5%
	Locally Advanced (Stage IIIB)		6	3.4%
	Locally Advanced (Stage IIIC)		5	2.8%
	Metastatic (Stage IV)		119	66.9%
ECOG performance status at the time of diagnosis of mBC^a^	0		63	35.4%
	1		96	53.9%
	2		18	10.1%
	3		1	0.6%
Adjuvant treatment received (history)	Yes		65	36.5%
	No		111	62.4%
	Don’t know		2	1.1%

*BC* breast cancer; ECOG: Easter Cooperative Oncology Group; mBC: metastatic breast cancer

^a^Definition of ECOG performance statuses; 0: Fully active, able to carry on all pre-disease performance without restriction; 1: Restricted in physically strenuous activity but ambulatory and able to carry out work of a light or sedentary nature, e.g. light house work, office work; 2: Ambulatory and capable of all self-care but unable to carry out any work activities. Up and about more than 50% of waking hours; 3: Capable of only limited self-care, confined to bed or chair more than 50% of walking hours

#### Treatment patterns

**Fig. 1:**
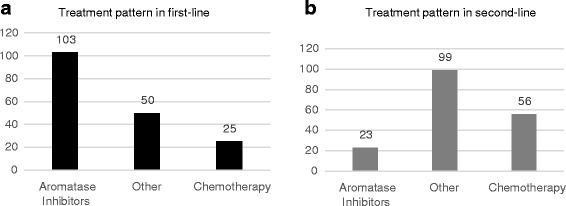
Treatment patterns in first (panel **a**) and second line (panel **b**) -*n* = 178; from chart review. Aromatase inhibitors: anastrozole, letrozole, exemestane and anastrozole + exemestane. Chemotherapy: capecitabine, docetaxel, paclitaxel, paclitaxel + carboplatin, docetaxel + cyclophosphamide, 5 fluorouracil, carboplatin, carboplatin + gemcitabine, cyclophosphamide + doxorubicin, docetaxel + carboplatin, goserelin, nab- paclitaxel. Other: tamoxifen, fulvestrant, everolimus + exemestane, anastrozole + paclitaxel, anastrozole + fulvestrant, anastrozole + tamoxifen, anastrozole + docetaxel, bevacizumab, letrozole + fulvestrant, anastrozole + anthracycline + cyclophosphamide, anastrozole + paclitaxel + anthracycline, bevacizumab + anastrozole + tamoxifen, everolimus, everolimus + letrozole, everolimus + tamoxifen, exemestane + carboplatin, letrozole + zoledronic acid, letrozole + paclitaxel, tamoxifen + goserelin, vinorelbine + lapatinib, lapatinib, toremifene-citrate. Note: “Other” refers to other treatments than aromatase inhibitors and chemotherapy agents

Aromatase inhibitors (anastrozole, letrozole and exemestane) were prescribed for the majority of patients in first line (103 out of 178; 58%) and for only 13% of patients in second line (23 out of 178). Other therapies (e.g. tamoxifen, fulvestrant or everolimus), or aromatase inhibitors combined with chemotherapy was given to 28% (50 out of 178) of patients in first line and 55.6% (99 out of 178) of patients in second line. Among the 50 patients receiving other therapies in first line, 43 patients were treated by endocrine therapy and the seven remaining patients were treated by everolimus (*n* = 4), bevacizumab (*n* = 2) and lapatinib (*n* = 1). Chemotherapy only was administered in 14.0% (25 out of 178) of patients in first line and in 31.5% (56 out of 178) of patients in second line (Fig. 1). Patients receiving chemotherapy in first line were more likely to have visceral disease than patients receiving other therapies (79.2% vs. 49.7%, *p* = 0.0071).

**Fig. 2:**
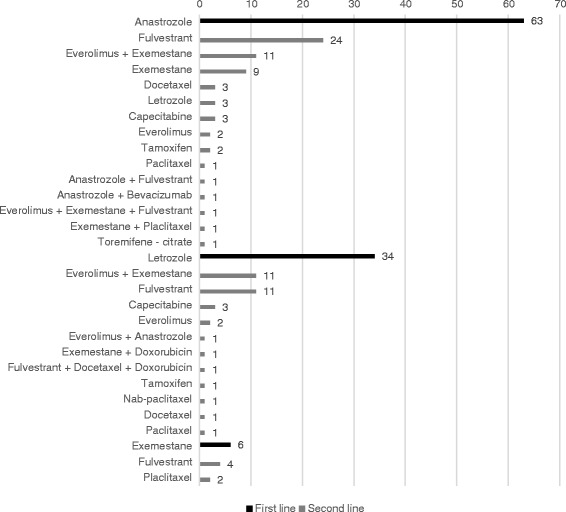
Treatment patterns after aromatase inhibitors in first line (*n* = 103) – from chart review. Data show absolute frequencies of treatments received in second line for patients that received anastrozole (*n* = 63), letrozole (*n* = 34) or exemestane (*n* = 6) in first line

As shown in Fig. 2, the most frequently described treatment in first line was the aromatase inhibitor anastrozole (63 out of 178; 35.4% patients). Thirty-eight percent (*n* = 24) of patients receiving anastrozole in first line switched to fulvestrant in second line. Letrozole was administered in first line for 19.1% (34 out of 178) of patients. For these patients, the everolimus plus exemestane treatment combination and the fulvestrant endocrine therapy were the most frequently given subsequent treatment in second-line (for both everolimus + exemestane and fulvestrant: 32.4%; 11 out of 34). Exemestane was prescribed for only 3.4% of patients in first line (6 out of 178).

#### Disease progression

**Fig. 3:**
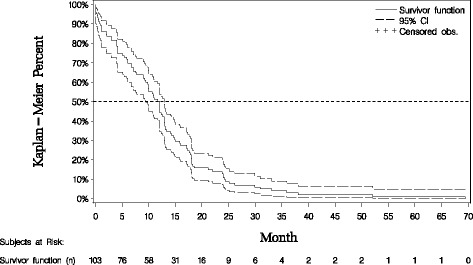
Time to progression on first line therapy with aromatase inhibitors – from chart review. Survivor function at 2 months: 0.845 / Survivor function at 3 months: 0.816 / Survivor function at 5 months: 0.747; median time to progression: 12.0 months

In first line, patients treated with chemotherapy progressed earlier (median time to disease progression: 7.9 months; 95% CI: 6.0 to 8.3) than those treated with aromatase inhibitors (12.0; 95% CI: 10.0 to 13.1) or other treatments including combination therapies of aromatase inhibitors and chemotherapy (11.9; 95% CI: 7.0 to 17.3), although the difference was not statistically significant – see Table 2. The Kaplan Meier curve for time to disease progression (TTP) in the subset of patients receiving aromatase inhibitors in first line (*n* = 103) shows that the probability of being progression-free at 3 and 5 months after start of first line therapy was 81.6% and 74.7% respectively (Fig. 3). In second line, median PFS was 7.3 (95% CI: 5.1 to 11.2), 7.4 (95% CI: 5.7 to 8.4) and 8.1 (95% CI: 7.0 to 12.0) months for patients receiving chemotherapy, aromatase inhibitors and other treatments, respectively. On third line treatment, the median PFS was higher for patients treated by chemotherapy compared with those treated by aromatases inhibitors and other treatments: 9.0 months for chemotherapy, 8.0 months (95% CI: 3.4 to 12.0) for aromatase inhibitors and 5.2 months (95% CI: 4.0 to 14.1) for other treatments.

**Table 2:** Time to disease progression and PFS by drug category and treatment line – from chart review

Variable	First-line treatment	*n*	# Censored obs.^a^	Median (months)	95% confidence interval		*p*-value^b^
					Lower limit	Upper limit	
Time to disease progression on first-line therapy (months) from start of first-line therapy	Aromatase inhibitors	103	8	12.0	10.0	13.1	
	Chemotherapy	25	13	7.9	6.0	8.3	
	Other	50	8	11.9	7.0	17.3	0.3563
Progression- free survival on second line therapy (months) from start of second-line therapy	Aromatase inhibitors	103	16	7.4	5.7	8.4	
	Chemotherapy	25	8	7.3	5.1	11.2	
	Other	50	19	8.1	7.0	12.0	0.1047
Progression- free survival on third-line therapy (months) from start of third-line therapy	Aromatase inhibitors	55	23	8.0	3.4	12.0	
	Chemotherapy	11	6	9.0	1.4	NE	
	Other	20	7	5.2	4.0	14.1	0.9176

*PFS* Progression-free survival; *NE* Not Estimable

^a^Censored patients are patients who have not had an event of disease progression, either because they dropped out from the trial for reasons other than disease progression or because they had not progressed when data were cut-off. ^b^ log rank test

As per inclusion criteria, 80% of patients were required to be alive at data abstraction. Accordingly, the Kaplan Meier estimate for the probability of survival at 24 months after start of first line treatment was 87.6%.

#### Reasons for treatment discontinuations

The most frequently reported primary reason of treatment discontinuation was efficacy in terms of disease progression and this was true for agents received in all the three first treatment lines. Disease progression accounted for 76.4% (168 out of 220 agents) of reasons reported in first line, 71.6% (169 out of 236 agents) of reasons in second line, and 50.4% (57 out of 113 agents) of reasons in third line (Table 3).

**Table 3:** Primary reasons for treatment discontinuation – from chart review

Primary reason for treatment discontinuation	First line (*N* = 220^a^)	Second line (*N* = 236^a^)	Third line (*N* = 113^a^)
	*n*, (%)	*n*, (%)	*n*, (%)
Disease progression	168 (76.4%)	169 (71.6%)	57 (50.4%)
Total completion of treatment	31 (14.1%)	30 (12.7%)	7 (6.2%)
Toxicities or side effects	14 (6.4%)	8 (3.4%)	7 (6.2%)
Patient choice (non-financial)	4 (1.8%)	8 (3.4%)	5 (4.4%)
Financial reasons	1 (0.5%)	1 (0.4%)	1 (0.9%)
Drug resistance	2 (0.9%)	0 (0.0%)	1 (0.9%)
Patient physical status	0 (0.0%)	2 (0.8%)	1 (0.9%)
Death related to mBC	0 (0.0%)	5 (2.1%)	3 (2.7%)
Death not related to mBC	0 (0.0%)	2 (0.8%)	1 (0.9%)
Treatment still ongoing	0 (0.0%)	11 (4.7%)	30 (26.5%)

There were more agents than patients per line (e.g. 220 agents vs. 178 patients in first line) as physicians were asked to provide reasons of discontinuation for every single agent rather than for every therapy

*mBC* metastatic breast cancer

^a^Number of agents

Across all treatment lines, bone pain and fatigue were reported as the most frequent symptoms associated with disease progression. Bone pain was reported for 54.4% (*n* = 81) of the 149 patients that progressed in first line and for 56.9% (*n* = 74) of the 130 patients that progressed in second line. Fatigue was reported for 41.6% (*n* = 62) of patients in first line and 43.8% (*n* = 57) of patients in second line.

#### Characteristics of patients early discontinuing aromatase inhibitors

Characteristics of patients who discontinued treatment with aromatase inhibitors earlier than 5 months after treatment initiation (*n* = 26) were not significantly different from the 76 patients who discontinued treatment later than 5 months (Table 4). However, early treatment discontinuation was less likely in patients receiving letrozole than in patients receiving anastrozole or exemestane (*p* = 0.0036).

**Table 4:** Treatment discontinuation on first-line therapy with aromatase inhibitors before vs. after 5 months - from chart review

Variable	Level	Treatment discontinuation ≤ 5 months (*N* = 26)		Treatment discontinuation > 5 months (*N* = 76)		*p*-value
Stage at diagnosis	Early (Stage IA, IB, IIA, IIB)	5	19.2%	12	15.8%	0.4740^b^
	Limited Regional (Stage IIIA)	2	7.7%	1	1.3%	
	Locally Advanced (Stage IIIB)	0	0.0%	1	1.3%	
	Locally Advanced (Stage IIIC)	0	0.0%	2	2.6%	
	Metastatic (Stage IV)	19	73.1%	60	78.9%	
ECOG performance status at the time of diagnosis of mBC ^a^	0	9	34.6%	28	36.8%	0.8021
	1	13	50.0%	40	52.6%	
	2	4	15.4%	8	10.5%	
	3	0	0.0%	0	0.0%	
De novo / relapse	De novo	19	73.1%	61	80.3%	0.5575^b^
	Relapse from adjuvant	7	26.9%	14	18.4%	
	Don’t know	0	0.0%	1	1.3%	
Adjuvant treatment received	Yes	8	30.8%	17	22.4%	0.4328^b^
	No	18	69.2%	59	77.6%	
	Don’t know	0	0.0%	0	0.0%	
Aromatase inhibitors received in first-line	Anastrozole	16	61.5%	46	60.5%	0.0036^b^
	Exemestane	5	19.2%	1	1.3%	
	Letrozole	5	19.2%	29	38.2%	
Visceral disease	Yes	15	57.7%	31	40.8%	0.1349
	No	11	42.3%	45	59.2%	

*mBC* metastatic breast cancer

One patient using aromatase inhibitors in first-line was excluded due to treatment discontinuation (patient choice) at 3 months

^a^Definition of ECOG performance statuses; 0: Fully active, able to carry on all pre-disease performance without restriction; 1: Restricted in physically strenuous activity but ambulatory and able to carry out work of a light or sedentary nature, e.g. light house work, office work; 2: Ambulatory and capable of all self-care but unable to carry out any work activities. Up and about more than 50% of waking hours; 3: Capable of only limited self-care, confined to bed or chair more than 50% of walking hours

^b^Exact Fisher test

### Physician survey

#### Physician characteristics

Physicians had treated on average 30 pre- and 50 post-menopausal mBC ER+ HER2- patients in the past 6 months, respectively. Seventy-two of the 103 physicians were working in a clinic-based practice or had an office, whereas 13 physicians provided care in a community hospital based practice (25, 23, 25 and 30 physicians of the 103 physicians were based in North East, Middle-West, West and South, respectively). The remaining 18 physicians were from university hospitals, tumour centers or an NCI-designated cancer center.

According to the physicians surveyed, on average 32% of all their newly diagnosed post-menopausal BC patients had metastatic disease at initial diagnosis of BC. Thirty-three percent of patients were diagnosed at an early stage of BC (stage I or II) while the mean percentage of patients diagnosed at stage IIIA, IIIB or IIIC was 13%, 11% and 12% respectively.

#### Treatment goals and treatment selection

Physicians were asked about the three most important treatment goals for first-line therapy. As is shown in Table 5, prolonging life was deemed the most important reason for treatment (58.3%). The most frequently mentioned responses for the second most important reasons of treatment were quality of life improvement/preservation (23.3% for quality of life improvement and 19.4% for quality of life preservation), respectively. For the third most important reason, it was symptom relief (24.3%).

**Table 5:** Goal of treatment – from physician survey (*n* = 103)

	First Rank		Second Rank		Third Rank	
Treatment Goal	*n*	*%*	*n*	*%*	*n*	*%*
Prolongate life	60	58.3%	13	12.6%	14	13.6%
Stabilize disease	1	1.0%	12	11.7%	11	10.7%
Preserve Quality of life	11	10.7%	20	19.4%	12	11.7%
Delay chemotherapy	5	4.9%	6	5.8%	18	17.5%
Symptom relief	5	4.9%	18	17.5%	25	24.3%
Tumour shrinkage	9	8.7%	10	9.7%	7	6.8%
Improve quality of life	12	11.7%	24	23.3%	16	15.5%

Physicians used hormonal therapies (aromatase inhibitors or tamoxifen) for around half of patients (51.9%) in first-line, followed by chemotherapy which was given to 17.6% of patients. In second-line, a fifth of patients received either oral hormonal therapy (21.1%), exemestane plus everolimus (22.4%) or other hormonal therapy (21.7%), respectively, and 25.6% of them were treated with chemotherapy. In third line, treatment patterns become even more diverse but with more patients receiving chemotherapy (35.8%): around 21.3% of patients received exemestane with everolimus and 12.6% and 16.3% of patients received oral hormonal and other hormonal therapy, respectively (Table 6).

**Table 6:** Treatment selection: Proportion of therapies for post-menopausal ER+, HER2- metastatic BC patients used in first-line, second-line and third-line in the past 6 months – from physician survey (*n* = 103)

	First Line		Second Line		Third Line	
Treatment	Mean	SD	Mean	SD	Mean	SD
Oral hormonal therapy (e.g., tamoxifen, aromatase inhibitors)	51.91%	32.48	21.14%	22.40	12.55%	15.02
Other hormonal (e.g., fulvestrant)	11.68%	17.54	21.66%	23.76	16.32%	19.98
Chemotherapy	17.55%	18.43	25.58%	23.12	35.83%	27.27
Exemestane plus Everolimus	9.50%	14.44	22.38%	24.41	21.26%	23.71
Avastin plus chemotherapy	4.19%	9.73	4.91%	10.44	6.17%	14.42
Clinical trial	1.90%	6.16	2.34%	6.20	3.89%	9.27
Other	3.25%	-	2.00%	-	3.97%	-

*BC* Breast cancer; *SD* standard deviation

#### Expectations on and limitations of treatment success

Physicians were asked to provide their experience with duration of PFS and OS for current treatments. In their experience, duration of PFS for the first treatment in ER+ HER2- mBC patients is around 13 months. Physicians also reported that they consider on average an increase of 7.4 months (median 6 months) as the minimum clinically meaningful improvement in progression-free survival over current standard of care for a new treatment of post-menopausal ER+, HER2- mBC. In terms of overall survival from start of first treatment, physicians’ current experience was close to 29 months or 2.4 years.

The physicians were asked to list the main treatment limitations of current treatments on a scale from 1 to 5 (5: very substantial, 4: substantial, 3: moderately, 2: somewhat, 1: not at all substantial). The main limitations reported were efficacy and safety/tolerability of treatments (Table 7). Focusing on aromatase inhibitors only, efficacy was still the limitation that most physicians perceived as either substantial or very substantial (46.6%), but an equal proportion also considered drug resistance as a substantial or very substantial treatment limitation (Table 8).

**Table 7:** Limitations of treatment success in first-line ER+, HER2- mBC patients - overall by analysis of categories – from physician survey (*n* = 103)

	Not at all substantial		Not at all substantial		Moderately substantial		Substantial		Very substantial	
	*n*	%	*n*	%	*n*	%	*n*	%	*n*	%
Efficacy	7	6.8%	12	11.7%	25	24.3%	25	24.3%	34	33.0%
Safety/Tolerability	5	4.9%	22	21.4%	27	26.2%	35	34.0%	14	13.6%
Adherence	16	15.5%	29	28.2%	22	21.4%	27	26.2%	9	8.7%
Financial cost of treatments	2	1.9%	27	26.2%	30	29.1%	35	34.0%	9	8.7%

*mBC* metastatic breast cancer

**Table 8:** Limitations of treatment success in first-line ER+, HER2- mBC patients - aromatase inhibitors only – from physician survey (*n* = 103)

	Not at all substantial		Not at all substantial		Moderately substantial		Substantial		Very substantial	
	*n*	*%*	*n*	*%*	*n*	*%*	*n*	*%*	*n*	*%*
Efficacy	4	3.9%	8	7.8%	43	41.8%	25	24.3%	23	22.3%
Safety/Tolerability	16	15.5%	24	23.3%	23	22.3%	29	28.2%	11	10.7%
Drug resistance	1	1.0%	12	11.7%	42	40.8%	41	39.8%	7	6.8%
Adherence	13	12.6%	23	22.3%	33	32.0%	24	23.3%	10	9.7%
Financial cost of treatments	14	13.6%	24	23.3%	28	27.2%	28	27.2%	9	8.7%

*mBC* metastatic breast cancer

## Discussion

The present study investigated patient characteristics, treatment patterns and time to disease progression through a retrospective review of medical records from ER+/HER2- mBC patients in the US and also assessed characteristics of patients experiencing early treatment discontinuation. Furthermore, the empirical real-world data from the chart review were supplemented, for some aspects, by a physician survey conducted among the 103 physicians who extracted the data.

The chart review data showed that following mBC diagnosis, the majority of patients received endocrine therapy (82%, including 58%(103/178) of aromatase inhibitors and 24% (43/178) of other ET) as a first-line treatment, with the aromatase inhibitors anastrozole and letrozole being the most frequently prescribed therapies. However a significant proportion (14%) of patients received chemotherapy (including chemo monotherapy or chemo combination therapies) as the first-line treatment. The potential reasons for chemotherapy use in first line could be concerns about endocrine resistance or the higher frequency of visceral metastases among these patients [17].

These findings are consistent with previous studies examining treatment patterns in ER+/HER2- mBC patients: Macalalad et al. (2015) [15] who described treatment patterns in post-menopausal women with HR+/HER2- metastatic breast cancer in a US retrospective chart review, presented first line treatment patterns with 84% of patients treated with endocrine therapy (or treatment in combination with ET), 14% of them with chemotherapy (monotherapy or combination of chemotherapy agents) and 2% with other therapies (*n* = 144). Xie et al. performed a chart review in the US for the same population of patients, they showed that 87% and 13% of them were under endocrine therapy and chemotherapy respectively at baseline in patients with a single metastasis [16].

The median time to progression for patients included in this chart review who were treated with aromatase inhibitors in first line was 12 months. This is consistent with estimates from previous studies which reported a time to progression between 8.2 months and 13.1 months for anastrozole used in first line [18–20]. With a median of 8 months, time to progression during first-line therapy for patients receiving chemotherapy was markedly shorter. Median PFS on second line therapy was shorter than on first line and did not significantly differ by type of therapy received in first line. Regarding patients treated by aromatase inhibitors in first line, the median time to progression in third line was similar for those treated either by chemotherapy or aromatase inhibitors (9 months and 8 months respectively). This last clinical outcome is consistent with the NCCN guideline who recommends chemotherapy after three sequential endocrine therapy regimens. However chemotherapy is associated with important side effects which impair patient quality of life.

The overall findings of the study highlight a quantitative unmet need for more effective treatments which delay disease progression and improve survival outcomes while maintaining quality of life. This was also expressed by the physicians, who participated in the survey, stating that prolongation of life, delaying deterioration in symptoms, and preserving or improving quality of life represent the most important treatment goals for them. Also, the majority of physicians considered limited efficacy as the most substantial limitation of currently available treatments. Finally, the survey also indicated that physicians consider an increase in progression-free survival of 7 months or more as clinically relevant to patients.

The chart review observed that 74.5% of patients treated with aromatase inhibitors in first line have not experienced disease progression after 5 months, while 25.5% of patients did. It was hypothesised that this group of early progressors represents a subgroup of patients who are early non-responders to aromatase inhibitors and who ideally should be prescribed another treatment after progression or ideally should be identified early so that early progression can be prevented by using a different treatment. The current study was not able to identify specific clinical or patients characteristics that could be predictive of early non-responders, mainly due to the low numbers of patients available for this analysis. However, there were fewer patients treated with letrozole in first line who discontinued before 5 months as compared to those continuing beyond 5 months (5 (19.2%) vs. 29 (38.2%), *p* = 0.0036). This might be related to potentially better efficacy of letrozole in comparison with other aromatase inhibitors. In previous in vivo measurement studies, letrozole demonstrated a better biochemical efficacy with a greater suppression of plasma oestrogen levels than anastrozole at clinical doses [21, 22]. However, a 5-year comparative efficacy study of letrozole and anastrozole in postmenopausal hormone receptor-positive early BC didn’t demonstrate any significant difference in disease free progression and survival rates [23].

Despite clear guidelines on the preferential use of multiple lines of endocrine therapy versus chemotherapy in advanced ER+ BC, a review of practice patterns using data from 2004 to 2010 have shown that these therapies were not being used as recommended [15]. The current study provides a more recent review of practice patterns in a rapidly evolving treatment landscape using data from 2008 to 2014. The study further adds to current knowledge on real-world outcomes in mBC since previous studies did not report data on clinical outcomes such as time to progression [13, 15]. Also, similar chart review studies in mBC patients did not describe treatment patterns [14, 16] or evaluated the effectiveness of specific treatment options only [14, 24]. Finally, by providing both, quantitative data from a chart review and qualitative data from the accompanying physician survey, the study provides a comprehensive picture of treatment selection, clinical outcomes, treatment goals and current limitations of treatments as perceived by physicians and their patients.

Though many efforts were undertaken to overcome these, this study has limitations inherent to the retrospective nature of the chart review study, the descriptive nature of the statistical analyses and the subjective nature of the physician survey. It is further possible, that the results were confounded by potential factors that were not identified. A key limitation for analyses related to the early non-responders was the small sample size (*n* = 26) which may have led to us not being able to identify specific patient characteristics for this patient subgroup. Also, it must be kept in mind that inclusion criteria required patients to have completed at least two lines of therapy and that a quota for survival status was used to ensure that 80% of patients were still alive at the date of data abstraction. While this ensured that there are sufficient data on treatment patterns in first- and second line, it may bias results towards “healthier” or longer living patients in this population. For this reason, the analysis of OS must be considered with caution.

Despite these limitations the sample of physicians was representative of physicians treating mBC in the US and the current study provides important insights about real world outcomes for ER+ HER2 mBC patients and their current unmet medical need.

## Conclusion

This study provides new evidence on treatment patterns and real-world clinical outcomes for post-menopausal ER+ HER2- metastatic breast cancer patients in the US. The retrospective chart review revealed that a majority of 82% of patients received endocrine therapy as first-line treatment and showed that current therapies in ER+ HER2- mBC still result in a short time to progression in first line. In the accompanying survey, physician considered limited efficacy and tolerability as the main deficiencies of current treatments, and consider improvements of quality of life as an important treatment goal. The overall finding from this study highlight a quantitative unmet need for more effective treatments which delay disease progression and improve survival outcomes while maintaining quality of life.

## Abbreviations

ASCO: American Society of Clinical Oncology
BC: Breast Cancer
ECOG: Eastern Cooperative Oncology Group
ER +: Estrogen receptor-positive.
ET: Endocrine Therapy
HER2-: Human Epidermal Growth Factor Receptor 2 negative
IRB: Institutional Review Board
mBC: Metastatic Breast Cancer
NCCN: National Comprehensive Cancer Network
OS: Overall Survival
PFS: Progression-free Survival
TTP: Time to progression
US: United States
